# Extensive Sclerosing Mesenteritis of the Rectosigmoid Colon Associated with Erosive Colitis

**DOI:** 10.1155/2009/176793

**Published:** 2009-04-12

**Authors:** C. Nobili, L. Degrate, R. Caprotti, C. Franciosi, B. E. Leone, F. Romano, M. Dinelli, Fr. Uggeri

**Affiliations:** ^1^Department of Surgery, San Gerardo Hospital, University of Milano-Bicocca, via Pergolesi 33, 20052 Monza, Milano, Italy; ^2^Department of Clinical Pathology, San Gerardo Hospital, University of Milano-Bicocca, via Pergolesi 33, 20052 Monza, Milano, Italy; ^3^Unit of Endoscopy, San Gerardo Hospital, University of Milano-Bicocca, via Pergolesi 33, 20052 Monza, Milano, Italy

## Abstract

Sclerosing mesenteritis is a rare, idiopatic, usually benign, inflammatory process of the mesenteric adipose tissue. The most common site of involvement is the small bowel mesentery. We present a case of sclerosing mesenteritis of the rectosigmoid colon as a cause of severe abdominal pain, abdominal obstruction, and ischemic colic mucosal lesions. Contrast enema, colonoscopy, angiography, and CT were the imaging modalities used. A 20 cm diameter, fibrotic mass causing extensive compression of rectosigmoid colon was found at laparotomy. Histological examination showed extended fibrosis, inflammatory cells infiltration, lipophages, and granulomas within the mesenteric adipose tissue associated with erosive colitis. Clinical presentation and treatment are discussed.

## 1. Introduction

Sclerosing mesenteritis (SM) is a rare, usually benign disorder that affects the mesenteric fat tissue. This disease usually arises from the mesentery of the small bowel and rarely involves the mesocolon [1, 2]. SM can express microscopically as predominantly fat tissue lesions (termed mesenteric panniculitis) or as predominantly fibrotic lesions (termed retractile mesenteritis). Clinical presentations and imaging patterns are nonspecific, so only the histopathologic analysis of SM masses provides a sound diagnosis. About the prognosis, SM can show both a favorable course and a so much extended fibrotic process that can produce medical or surgical urgencies. It is likely that a malignancy is associated to SM lesions, so, although medical therapies have been proposed, only surgical complete removal can solve complications, if present, can prevent unfavorable progress, and can avoid the risk of hidden metachronous cancers. We report the case of a male patient with sclerosing mesenteritis complicated by intestinal obstruction. The diagnosis could be performed only by anatomopathologic findings and SM was treated by surgical resection. Discussion is focused on aetiology, histology, natural history and treatment.

## 2. Case Report

A 59-year-old man complained of two-month history intermittent lower abdominal pain and constipation. About one year before, due to dyspeptic symptoms, an esophagogastroduodenoscopy was carried out showing jatal hernia. His past medical history included only acute myocardial infarction 13 years before. Laboratory tests were not contributory; tumor markers carcinoembryonicantigen, alpha-fetoprotein, and carbohydraticantigen 19.9 were negative. Barium enema demonstrated rugged mucosa and serrated contour with narrowing of the sigmoid colon by a probable extrinsic encasement. Then, a colonoscopy revealed reduction until 14 mm of calibre of the sigmoid colon, with rigid walls and aphtosis of the rectum. Histological examination of the endoscopic biopsies diagnosed ischemic colitis. Angiography of the celiac artery and of the mesenteric arteries showed no abnormalities.

**Figure 1:**
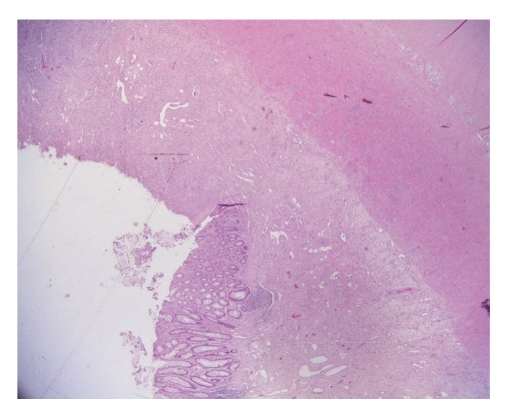
Idiopathic retractile sclerosing mesocolitis: inflammation of intestinal wall associated with erosion of mucosa (emathoxilyn-eosin 100x original magnification).

**Figure 2:**
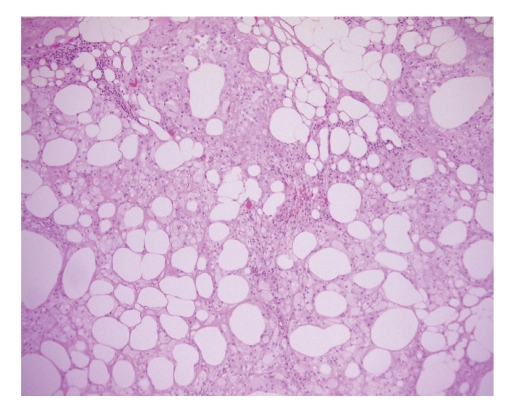
Idiopathic retractile sclerosing mesocolitis: lipid-laden foamy cells and chronic granulomatous inflammation of mesocolon (emathoxilyn-eosin 100x original magnification).

**Figure 3:**
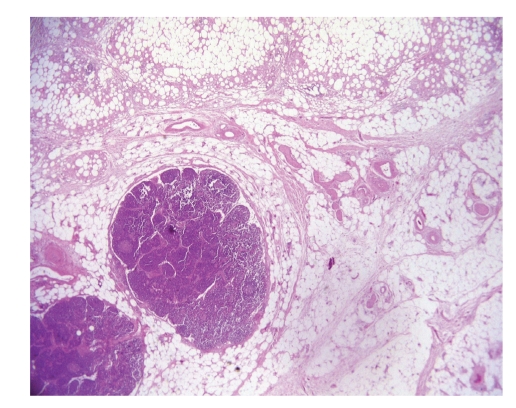
Idiopathic retractile sclerosing mesocolitis: aspecific reactive lymphadenopathy of pericolic nodes and lipophagic inflammation of mesocolon (emathoxilyn-eosin 100x original magnification).

Patient's symptoms got worse over the next month, mucous diarrhoea appeared and the patient presented to Emergency Room. On examination, he was afebrile. Physical examination of the abdomen revealed slight abdominal distension and, in the lower abdomen, a firm mass without tenderness, measuring approximately 10 cm in diameter, with mild pain on pressure, corresponding to the sigmoid colon. Rectal examination did not reveal anything abnormal. A computed tomography (CT) scan showed rare sigmoid divertucula and thickened walls (about 1.5 cm) of the rectosigmoid colon, determining mild luminal narrowing, without signs of perivisceral inflammation; liver was normal; there was not lymphadenopathy either ascites. During the third day of hospitalization, the patient developed obstructive symptoms with abdominal cramping and vomiting. A laparotomy identified a fibrous, partially necrotic, hyperaemic, 20 cm diameter mass in the mesocolon determining stenosis of the sigmoid colon and rectum, adherent to the parietal peritoneum. The mass was resected in toto with rectosigmoid colon; transanal anastomosis according to Knight-Griffen and temporary protective ileostomy were performed. Postoperative course was uneventful. Histological examination of surgical specimen revealed lipid-laden foamy cells and chronic granulomatous inflammation of mesocolon, associated with erosive colitis (Figures 1 and 2); these findings were consistent with idiopathic sclerosing mesocolitis; aspecific reactive lymphadenopathy of pericolic nodes (Figure 3).

Three months later, Gastrographin enema excluded intestinal fistula, so patient underwent ileostomy surgical closure. At two years follow-up patient is asymptomatic.

## 3. Discussion

Sclerosing mesenteritis (SM) is an uncommon nonneoplastic inflammatory process in the mesenteric fat that rises as a pseudotumor, usually involving the small bowel mesentery, the mesenteric fat and, less commonly, the mesentery of the large bowel. Young adults are mainly affected, more often men than women; about 2-3:1 [3–6], and the incidence increases above age 50 [3], although also rare cases of children have been reported [7].

Several names are proposed for this disease, as SM, retractile mesenteritis, mesenteric nodular panniculitis, mesenteric Weber-Christian disease of the mesentery, and mesenteric lipodystrophy, but they represent histological variants of a single pathologic entity that differs only in its proportion of fibrosis, lipid-laden macrophages, adiponecrosis, and nonspecific inflammatory infiltration. Microscopic diagnostic findings are chronic inflammatory process of the mesentery, characterized by fibrosis, myofibroblasts, and inflammatory cells infiltration, degeneration of the fatty tissue or fat necrosis; aggregations of lipid-laden foamy macrophages are also present and they can be distributed in bands of variable width or in scattered areas [3, 8, 9]. Two patterns of expression are known: mesenteric panniculitis (if, at histology, the lesion is characterized more by inflammation and fat necrosis than by fibrosis) and retractile mesenteritis (when the lesion is characterized by predominant fibrosis) [1, 10]. Our patient showed predominant inflammation and fat changes rather than fibrosis. The lesions are usually single mass; multiple masses or diffuse thickening of the mesentery are less common [5, 9]. The present case involved the large bowel mesentery with a single mass.

There is lack of consensus about the etiology of SM. Several causes have been hypothesized, as ischemia, infections, previous trauma, autoimmune disorders, previous abdominal surgery, coexisting malignancies moslty urogenital or gastrointestinal lymphomas [1, 2, 10].

Establishing the diagnosis of SM is both a clinical and histologic challenge. Clinical presentations are nonspecific and in common with numerous other diseases. Patients with SM may present with intestinal obstruction or ischemia, abdominal pain or distension, abdominal mass, weight loss, fatigue, fever of unknown origin, protein-losing enteropathy with diarrea, and minimal change nephropathy [11–14]. SM may be totally asymptomatic, incidentally discovered on CT performed for other reasons. Some Authors described concomitant SM and bile ducts fibrosis simulating Klatskin's tumor [15], SM involving the pancreas mimicking pancreatic malignancy [6], and SM and inflammatory pseudotumor simulating gastric lymphoma or linitis plastica [16]. The duration of symptoms can range from days to 10 years (average 12 months) [9].

Differential diagnosis take into account mesenteric lipomas and liposarcoma, but radiographic tools (contrast studies and CT or MRI [17]), even if very useful in the detecting of the mass, can give only a suspicion of this pathologic entity, because they provide nonspecific appearances, so the diagnosis can only be obtained by histology. A pseudoascess resulting from a diverticular diseases could be ruled out because of the absence of complicated sigmoid diverticula and the absence of septic findings.

SM has three modes of progression: partial or complete resolution of the inflammatory process, a nonprogressive course, or an aggressive course, characterized by a progressive fibrosis. That can cause shortening of the mesentery and compression of mesenteric vessels and then thrombosis with secondary variceal bleeding, ischemia or life-threatening intestinal obstruction or ureteric obstruction with peritonitic or uremic status. In our case colonoscopy found ischemic mucosal lesions, probably due to the compression of terminal colic vessels by the fibrotic process. SM usually has a bening course with a favorable outcome [4, 9] and spontaneous resolution has been reported [6], but there are not identified prognostic factors to predict the kind of progression. It has been suggested that colonic forms have a more aggressive course and require surgical treatment more often than other forms [5].

There is not any specific treatment for SM. Many medical modalities—including corticosteroids, colchicines, immunosuppressive drugs, and progesterone—have been used with varying degrees of success [8, 11, 18–22]. We think that complete laparotomic surgical resection is mandatory in presence of complications as obstructions and vascular involvement, but surgery has to be attempted also in order to allow a definite diagnosis by the histology and to avoid the risk of coexisting malignancy, that is hard to rule out in case of endoscopic or laparoscopic biopsies.
